# Patent research in academic literature. Landscape and trends with a focus on patent analytics

**DOI:** 10.3389/frma.2024.1484685

**Published:** 2025-01-08

**Authors:** Cristian Mejia, Yuya Kajikawa

**Affiliations:** Institute for Future Initiatives, The University of Tokyo, Tokyo, Japan

**Keywords:** patent analytics, bibliometrics, text mining, tech mining, network analysis

## Abstract

Patent analytics is crucial for understanding innovation dynamics and technological trends. However, a comprehensive overview of this rapidly evolving field is lacking. This study presents a data-driven analysis of patent research, employing citation network analysis to categorize and examine research clusters. Here, we show that patent research is characterized by interconnected themes spanning fundamental patent systems, indicator development, methodological advancements, intellectual property management practices, and diverse applications. We reveal central research areas in patent strategies, technological impact, and patent citation research while identifying emerging focuses on environmental sustainability and corporate innovation. The integration of advanced analytical techniques, including AI and machine learning, is observed across various domains. This study provides insights for researchers and practitioners, highlighting opportunities for cross-disciplinary collaboration and future research directions.

## 1 Introduction

Patents serve as a repository of technical and commercial knowledge, and protect intellectual property, playing an important role in promoting technological progress, business development, and innovation. These legal documents grant inventors temporary exclusive rights over their creations, incentivizing the disclosure of technical information that might otherwise remain hidden as trade secrets (Carr, 1995). By allowing inventors to profit from their creativity while simultaneously inspiring further technological advances through the revelation of prior art, patents directly impact both scientific and economic development (Hall, 2007; Langinier and Moschini, 2002; Schankerman, 1998).

Despite the rapid increase in academic literature exploring patents or leveraging patent documents and data, a current and comprehensive overview that captures the landscape of patent research holistically has yet to emerge. Such a panoramic perspective is invaluable for several reasons. First, it can reveal emerging topical clusters and current research trends, guiding scientists and practitioners toward areas of growing relevance. Second, mapping the landscape of patent research reveals the role and potential applications of more specialized subfields like patent analytics. This insight allows researchers developing quantitative patent analysis methods to focus their efforts on domains that stand to benefit most from such techniques. Finally, an academic landscape may facilitate cross-pollination across traditionally siloed disciplines by exposing potential applications of patent analytics.

To address this gap, our study aims to provide a landscape of patent research within academic literature. By surveying scholarly literature, we uncover major research topics, identify their interrelationships, and track evolving trends over time. We pay particular attention to the distinct and complementary studies of patent analytics, which have grown increasingly important for understanding innovation and economic progress. Specifically, this article addresses the following research questions:

1. How is patent information used in academic research?
2. What are the current trends in patent research?
3. What is the role of patent analytics methods within the larger scope of patent research?

In this article, we refer to “patent research” as any study that employs and leverages patents or patent data in any form and for any purpose, while “patent analytics” is used with a narrower scope to refer to studies with a more systematized approach to the study of patents or patent data, especially when used to develop metrics or methodologies (Daim et al., 2006). Patent analytics offers valuable insights into technology development trends, key industry players, and competitive landscapes through various approaches and techniques designed to extract meaningful information from patent data.

Our study employs a combination of data extraction techniques and topic analysis methods, including citation network analysis of scholarly articles. We present an overview of the current landscape, focusing on research fronts characterized by recency, relevance, and rapid growth (Rotolo et al., 2015). We expect to contribute by providing a comprehensive map of the patent research landscape to guide future studies and collaborations, identify emerging trends and underexplored areas to inform research priorities and funding decisions, provide insights into the evolving role of patent analytics to enhance evidence-based strategic planning and innovation policies, and develop a framework for integrating diverse patent research methodologies to foster interdisciplinary approaches in both academia and practitioners.

The remainder of this article is structured as follows: first, we explore the relevance of patents and scholarly patent research in general, while covering previous efforts in mapping the field of patent analytics. The methods section details our data extraction and analysis techniques. In the results section, we present our findings on the current landscape of patent research, with a focus on emerging trends and key areas of development. We conclude by discussing future directions for interdisciplinary research and shifts in methodological approaches within the field of patent research and analytics.

## 2 Previous literature

The academic interest in patent data spans several decades, evolving from early information retrieval systems to sophisticated analytical approaches. In the 1950s, pioneering work by Mooers (1952) laid the foundation for patent retrieval systems, initially focusing on searching metadata fields such as author, title, and keywords. As technology advanced, the scope expanded to include full-text analysis of patent documents in the 1960s and 1970s.

The 1970s and 1980s marked a significant shift in patent research, with scholars beginning to use patent statistics as a proxy for innovation and technological change. Soete (1979) and Pavitt (1985) examined the relationship between research and development (R&D) investment and patent counts at the national level, finding significant correlations. Other studies explored patenting patterns across countries and industries to understand differences in innovative activity (Evenson, 1984; Schiffel and Kitti, 1978; Sláma, 1981). At the firm level, Pakes and Griliches (1980) conducted one of the first systematic analyses of the relationship between R&D and patenting, finding a strong cross-sectional relationship but weaker time-series correlations.

A landmark contribution came from Trajtenberg (1990), who studied the computed tomography scanner industry. By combining patent data with market information, Trajtenberg demonstrated that while raw patent counts correlated poorly with social value creation, citation-weighted patent counts showed a strong correlation (around 0.75) with total social welfare created. This finding has been corroborated by subsequent studies, such as Harhoff et al. (1999) and Hall et al. (2005), establishing the importance of patent citations as indicators of economic and technological significance.

The analysis of patent citation networks emerged as a distinct field of study in the latter half of the 20th century. Early work by de Solla Price (1965) highlighted the importance of citation analysis in understanding scientific and technological development. The 1980s and 1990s saw the formalization of quantitative approaches, with Narin (1994) introducing various patent metrics for the study of Innovation. The release of the NBER Patent Citations Data File in 1990 provided researchers with a comprehensive dataset, spurring further studies on knowledge spillovers and innovation diffusion (Hall et al., 2001).

More recently, patent analytics has expanded its applications across various domains of technology management and innovation policy. Key areas include competitive intelligence, technology forecasting, R&D planning, merger and acquisition analysis, and policy evaluation. The exponential growth in global patent data, with 2022 alone estimated at 3.46 million patent applications worldwide (WIPO, 2023), has needed the development of more sophisticated and automated methods for analysis. Thus, the field has benefited from the integration of advanced techniques such as text mining, natural language processing, network analysis, and machine learning. This plurality of methodologies and scopes has led to the emergence of various terms describing the field, like patent bibliometrics (Narin, 1994), patinformatics (Trippe, 2003), and technology mining or tech mining (Porter, 2004), each with nuanced scopes and target applications, reflecting its multidisciplinary nature.

The use of patents by academics has been surveyed in the past, with the work of Basberg (1987) being an early example. This survey focused on the use of patents to measure technological change. Scholars at the time were concerned with the use of patent citations, finding “important” patents, and benchmarking innovation across regions. A comprehensive survey by Griliches (1990) reviewed several decades of research on patent statistics as economic indicators. He examined multiple data sources, including patent counts, renewal data, and stock market valuations. His survey synthesized evidence from studies using the U.S. Patent Office data, European patent renewal information, and firm-level R&D expenditure data highlighting critical measurement challenges, including the highly skewed distribution of patent values and variations in patenting propensity across industries and time. Griliches' synthesis helped establish methodological frameworks for evaluating patent quality and understanding the limitations of patent statistics as innovation indicators. More recent efforts have adopted computer-assisted methods to bring a more systematized understanding of the field by using bibliometrics (Mejia et al., 2021). Mikova (2016) analyzed Global TechMining conference proceedings from 2011 to 2015, identifying trends such as the integration of multiple approaches (e.g., bibliometrics, NLP, statistical analysis) and the use of novel data sources (e.g., web data, social media). Aristodemou and Tietze (2018) reviewed 57 articles on applying AI, machine learning, and deep learning to intellectual property data, categorizing them into knowledge management, technology management, economic value, and information extraction/management. The study found a growing interest in intellectual property (IP) analytics but called for more research on use cases and firm-level applications. Karata et al. (2024) analyzed 1,006 papers on “patent analysis,” revealing trough a descriptive approach that “technology” was the most common keyword and that top journals included “Technological Forecasting and Social Change” and “Information Processing & Management.” Hu et al. (2024) explored the foundations and frontiers of technology mining using co-citation analysis, bibliographic coupling, and content analysis of 277 articles. The study identified text analysis, bibliometrics, patent analysis, and strategic technology management as foundational areas, with technology topic analysis, roadmapping, component analysis, opportunity analysis, and management/decision support as frontier clusters.

While these previous studies offer valuable insights, they are limited in providing a comprehensive understanding of patent research given their narrower econometric or methodological focus. Our study aims to address these gaps by analyzing a larger and more recent dataset to capture the latest developments. It takes a holistic view of patent research, considering all aspects rather than focusing on specific methods or applications.

## 3 Materials and methods

The bibliographic data for this study was sourced from the Web of Science (WOS) Core Collection. To identify relevant articles, a topical search was conducted using the query TS = “patent^*^”, where the asterisk serves as a truncation symbol to accommodate variations of the term (e.g., patents). The search was performed without time constraints, retrieving articles from all available years in the database. Data were retrieved on May 31, 2024, yielding 103,738 articles.

While comprehensive, the query also retrieves articles unrelated to the target topic due to the various meanings of the term “patent”. In addition to referring to intellectual property documents, “patent” may be used as an adjective to denote open, unobstructed, or accessible, particularly in biomedical research. For example, there is extensive research on patent ductus arteriosus, a congenital heart defect (Schneider and Moore, 2006). Other deviating meanings include its use as a synonym for obvious, clear, or apparent. From a document retrieval standpoint, it may be tempting to generate a list of banned keywords (e.g., to be used with the NOT operator), but this would result in neglecting patent analytics papers on those alternative meanings [for instance, patent analysis of patent ductus research (Hsieh et al., 2004)]. Therefore, to focus on our target topic, citation networks were employed as both a data-cleaning mechanism and a means to extract thematic clusters.

Academic articles are positioned within a research field by citing previous related research. Articles that do not cite nor are cited by other articles were excluded from the study, as these are the papers that used the keyword “patent” without belonging to the patent research domain. A direct citation network was constructed, establishing linkages between articles when one cites the other (de Solla Price, 1965). Direct citation networks are known to surface research field taxonomies (Klavans and Boyack, 2017) and help identify research fronts (Shibata et al., 2008), making them suitable for long-term bibliometric research. However, this network would also contain papers in other fields of research, such as in biomedicine, that may cover other meanings of patents. To exclude these, thematic clusters were extracted, and after human inspection, unrelated clusters were pruned from the citation network.

**Figure 1:**
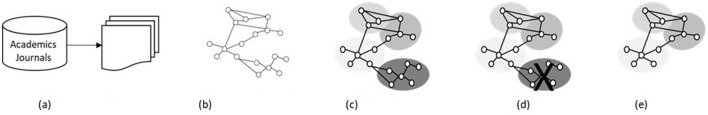
Overview of the methodology. **(A)** Data acquisition. **(B)** Citation network. **(C)** Clustering. **(D)** Remove unrelated clusters. **(E)** Cluster analysis.

Identifying topics from a citation network involves grouping nodes with denser connections compared to other groups. An optimal partition is achieved when the link density is higher at the intra-cluster level than the inter-cluster level, maximizing the network's modularity (Newman, 2006). We applied the Louvain method, a computationally efficient algorithm for partitioning large networks, to obtain the clusters (Blondel et al., 2008). For large networks, the first pass of the clustering algorithm may result in relatively large clusters. To obtain a more granular view, we applied the resolution limit theorem (Fortunato and Barthélemy, 2007) to further split clusters into subclusters, resulting in a topical hierarchy that facilitates the analysis, as the topics become smaller and more coherent. The authors named the clusters based on an assessment of the titles of the most connected articles, the most frequent keywords, and relevant metadata such as journal names, countries, or authors. During this step, the final cleaning was conducted, and unrelated clusters were removed from the study. Figure 1 represents a summary of the methodology.

Summary statistics of the publication years and citations received by the articles within each cluster and subcluster were calculated. Concretely, we apply metrics related to “size” being the number of documents; “relevance” the standardized cluster citations; “emergence” the average publication year; and “fast growth” the largest delta increase of publication counts within each subcluster over the past 10 years. These metrics are known to be useful in defining emerging research (Rotolo et al., 2015). Subclusters that are outliers in any of those metrics are separated for detailed descriptions.

Bibliographic data, including the full record and cited references, were exported as tab-delimited files from the WOS website. The dataset was processed using the statistical software R version 3.6.3 (R Core Team, 2019), with the igraph package version 1.2.5 (Csárdi et al., 2024) for network creation and clustering and the tm package version 0.7.7 for text processing (Feinerer et al., 2008). The citation network was visualized using the large graph layout (Adai et al., 2004), selected for its computational efficiency. The choice of layout has no impact on the research results.

## 4 Results

**Figure 2:**
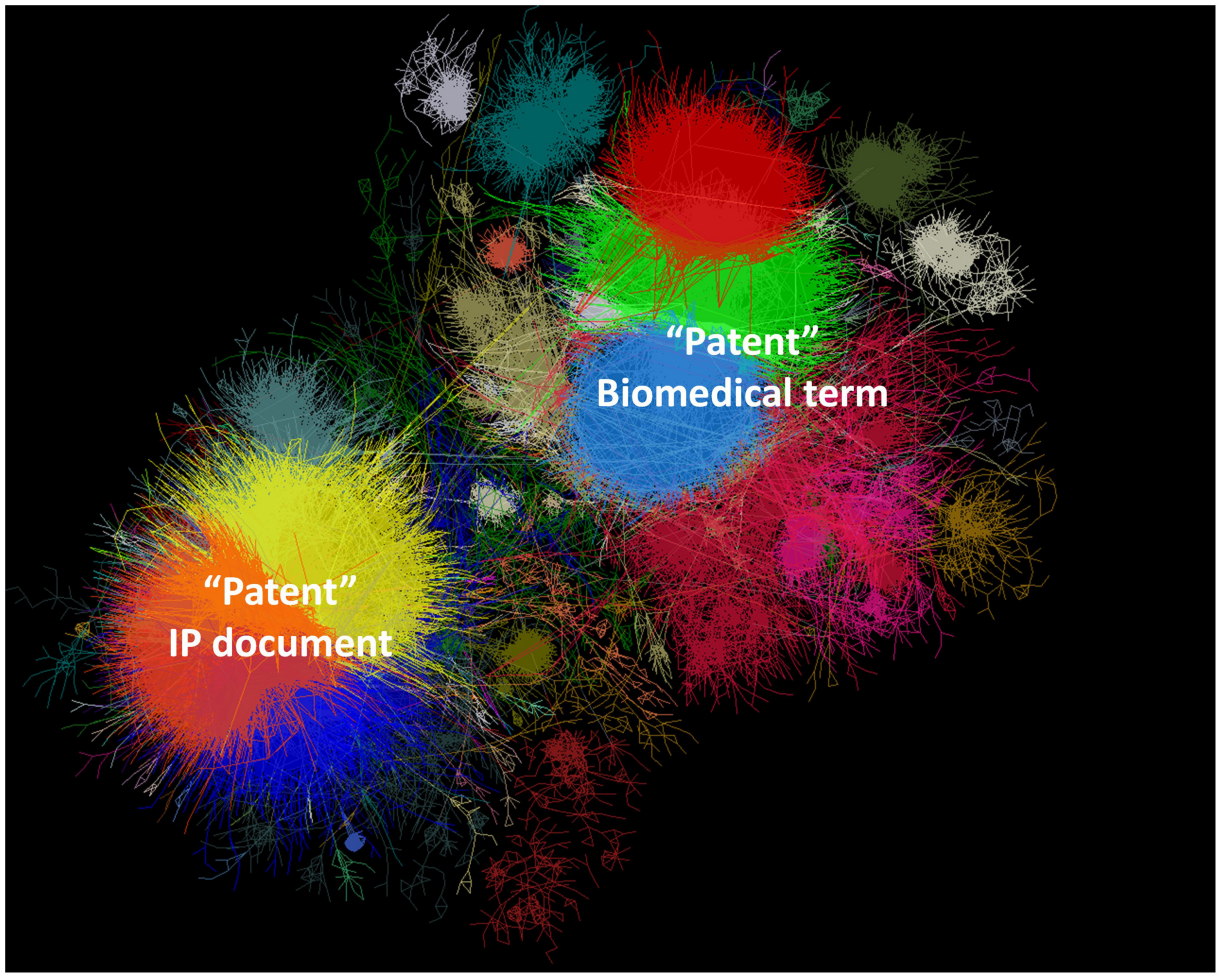
Citation network of patents. The divide between the use of “patent” in biomedicine, and as an IP document is apparent. This article focuses in the group of clusters in the bottom-left side.

From the original dataset, 53,668 articles used alternative meanings of the term “patent” that are not part of the core of patent research. These papers are disconnected from the main corpus of knowledge as they do not cite or receive citations from other patent-related literature. The rest of the articles compose the citation network of patents-related literature, covering 50,070 articles. Figure 2 shows the citation network forming a 2-group divide of clusters, one being those from biomedicine fields where patent is used in its medical meaning, and the other group of clusters studying patents as the IP document. Patent as the IP document has the largest share with 27,119 (54%) articles. In Figure 2, this group looks smaller due to its more cohesive nature, as the citations seem to be shared across the clusters in the group. In the remaining of this article, we refer as the citation network of patents research to this group of 27,119 documents and the results presented hereafter are based on this dataset.

The study of patents in academic literature has been steadily growing over the past decade, with an average 7% yearly increase in publications. The earliest records in the dataset of the divide date back to 1909, when Baekeland (1909) called for revisions to the United States (US) patent law. Since then, the trend of publications has been on the rise, with 2,139 publications in 2023 alone. The US and China have been the largest contributors to patent-related literature, with China consolidating its position as the country with the most publications since 2020. In 2023, more than 35% of publications came from China, while the US followed at 17%. The United Kingdom (UK) has consistently maintained its position as the third-largest contributor.

The study of patents spans across various fields, with Economics, Management, and Law being the most prominent. The journals that have been at the forefront of publishing patent-related literature include Research Policy, Reviews on Therapeutic Patents, Scientometrics, Technological Forecasting and Social Change, and Sustainability. Supporting figures for these descriptive statistics are presented in the Supplementary material.

### 4.1 Clusters

**Figure 3:**
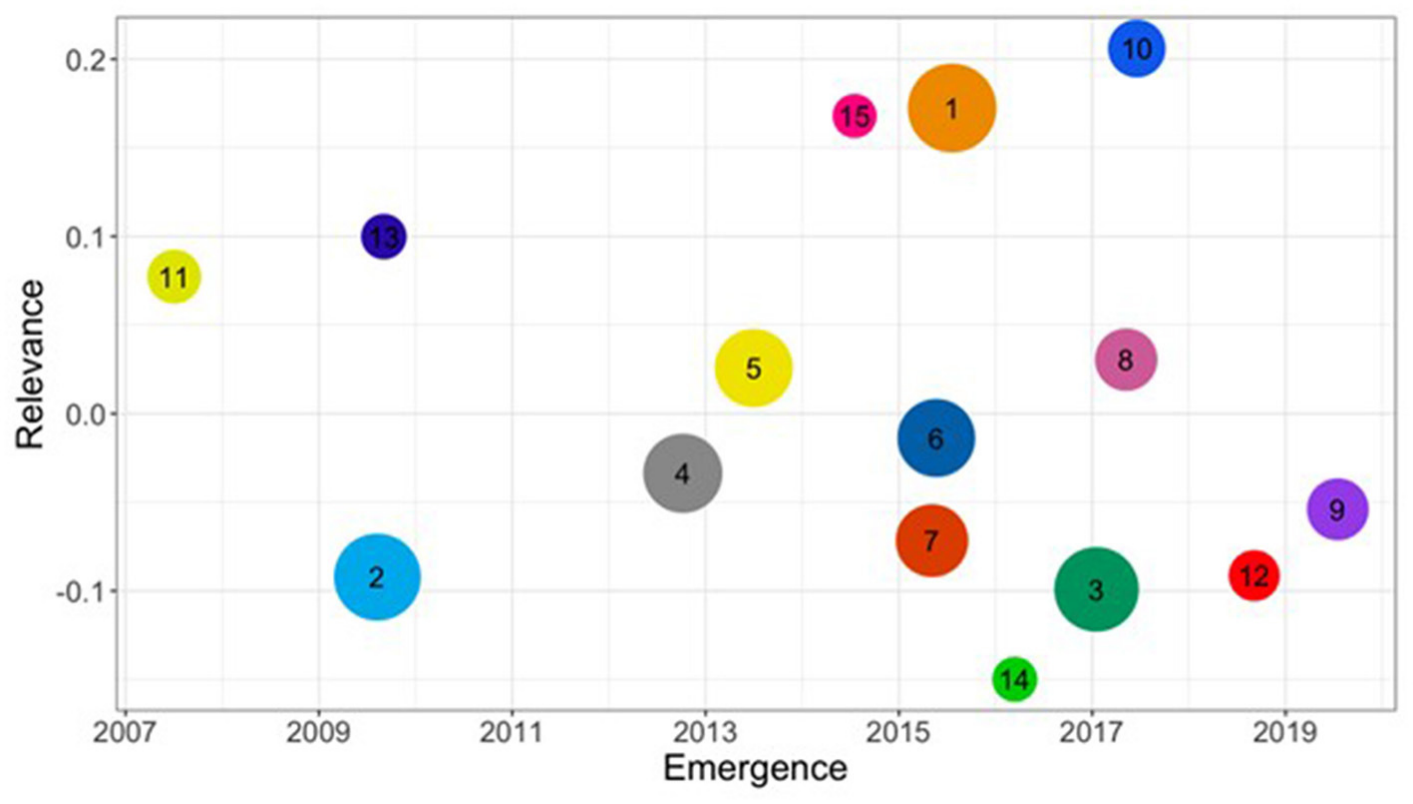
Patent research clusters. The size of each cluster represents the number of articles, the *x*-axis shows the average publication year (emergence), and the *y*-axis indicates the standardized average citations received (relevance). Each cluster is represented by a different color and numbered from the largest size.

Our analysis of the citation network yielded 15 distinct clusters, along with an additional grouping of very small clusters aggregated as “others”. These clusters represent the primary themes in patent research. Figure 3 presents a visual representation of these clusters, illustrating their relative size, emergence, and relevance. The underlying data is presented as Table 1.

**Table 1:** Patent research clusters summary.

**ID**	**Cluster**	**Articles**	**Ave. year**	**Ave. citations**
1	Patent analytics and innovation dynamics	3,660	2015.6	33.9
2	Patent systems and biomedical innovations	3,455	2009.6	13.2
3	Advanced methods in patent analytics and technology forecasting	3,183	2017.0	12.6
4	Economic implications of patent policies	2,659	2012.8	17.8
5	University-industry collaboration and knowledge transfer	2,545	2013.5	22.4
6	Strategic patent management and market dynamics	2,532	2015.4	19.3
7	Pharmaceutical patents and market access	2,059	2015.3	14.8
8	Corporate innovation and patent performance	1,284	2017.4	22.8
9	Environmental innovation and sustainable development	1,244	2019.5	16.2
10	Nanoparticle-based drug delivery systems	982	2017.5	36.5
11	Computational methods in drug discovery and patent analysis	773	2007.5	26.5
12	Patenting and traditional medicine in modern healthcare	627	2018.7	13.2
13	Emerging trends in drug development and therapeutics	402	2009.7	28.2
14	Patents and technology standards	382	2016.2	8.7
15	Carbonic anhydrase inhibitors research	345	2014.5	33.6
16	Others	987	2015.0	19.7

The field exhibits a clear evolution over time, with clusters spanning, on average, from 2007 to 2019. Larger clusters, such as “Patent Analytics and Innovation Dynamics” (1), “Patent Systems and Biomedical Innovations” (2), and “Advanced Methods in Patent Analytics and Technology Forecasting” (3), dominate the field, indicating areas of extensive research. The chart also highlights emerging trends, with clusters like “Environmental Innovation and Sustainable Development” (9) and “Patenting and Traditional Medicine in Modern Healthcare” (12) positioned toward the right, signifying more recent areas of focus. Matured research areas, represented by clusters such as “Computational Methods in Drug Discovery and Patent Analysis” (11), “Emerging Trends in Drug Development and Therapeutics” (13), and “Patent Systems and Biomedical Innovations” (2) are found on the left side of the chart. This also signals that, on average, the prevalent use of patent data in pharma and biomedical fields precedes that of innovation studies. The vertical axis reveals varying levels of citation impact, with clusters 1, 10, and 15 showing the highest relevance. Notably, “Patent Analytics and Innovation Dynamics” (1) stands out as a large, recent, and highly cited cluster, suggesting its dominant and influential role in current research. The lso captures a shift from general patent system and policy-related research toward more specialized and application-oriented topics over time. A granular view of the evolution in the vocabulary related to each cluster and their most frequent fields of research based on the Web of Science classification is offered in Figure A3 and Figure 7 in the Appendix, respectively.

### 4.2 Subclusters

**Figure 4:**
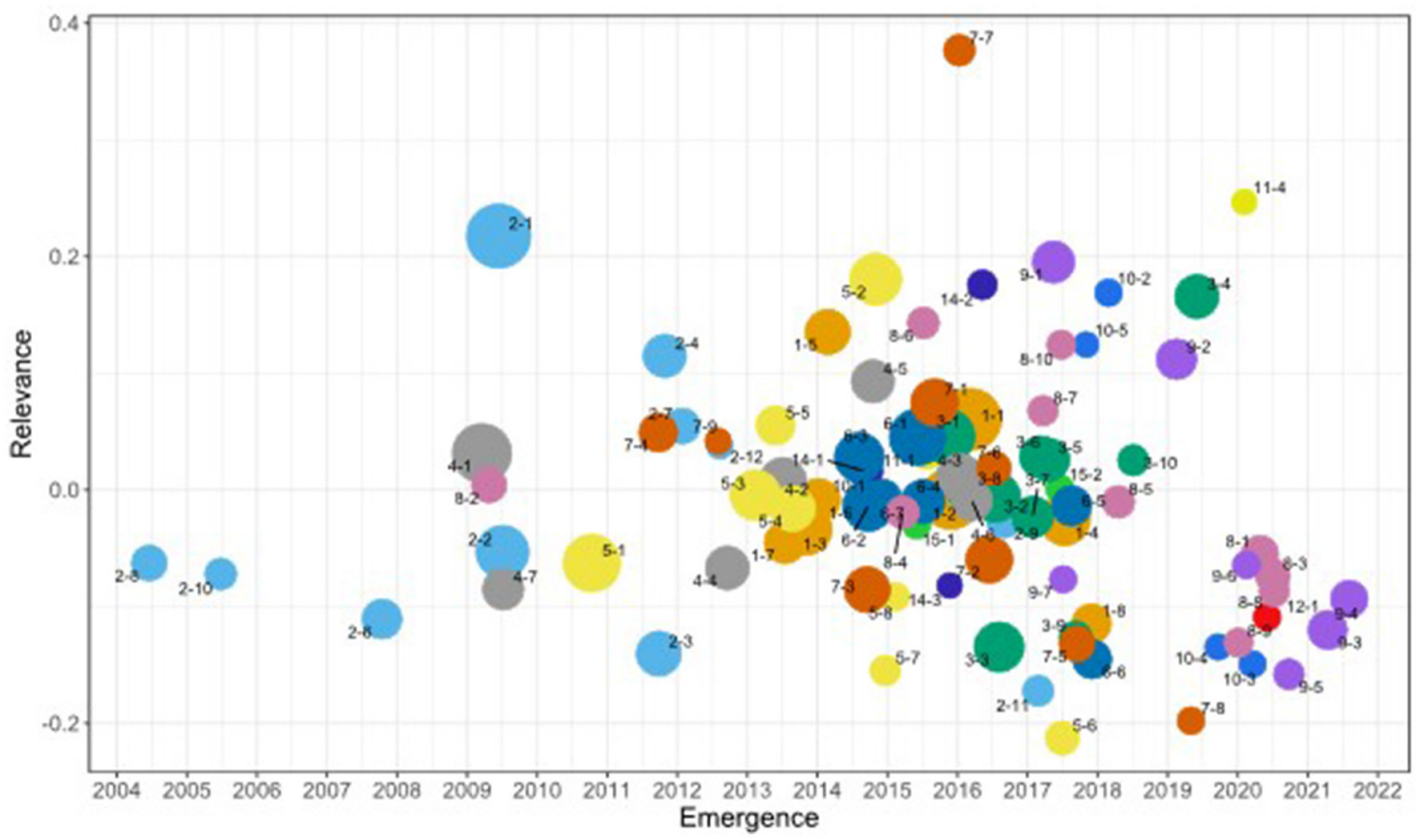
Patent research subclusters. The size of each subcluster represents the number of articles, the *x*-axis shows the average publication year (emergence) from 2004, and the *y*-axis indicates the standardized average citations received (relevance). Each cluster is represented by a different color and numbered from the largest size. Labels indicate subcluster codes.

We identified 93 distinct subclusters derived from the main clusters. These provide a more granular view of the research landscape within patent research. Figure 4 replicates Figure 3 at the subcluster level. The underlying data is available in the Table A1. The naming convention for subclusters follows a two-part code, where the first number represents the main cluster, and the second number indicates the subcluster's position within that main cluster. For instance, subcluster “1-2” denotes the second largest subcluster within main cluster one. Subclusters are ordered by size within each main cluster.

**Figure 5:**
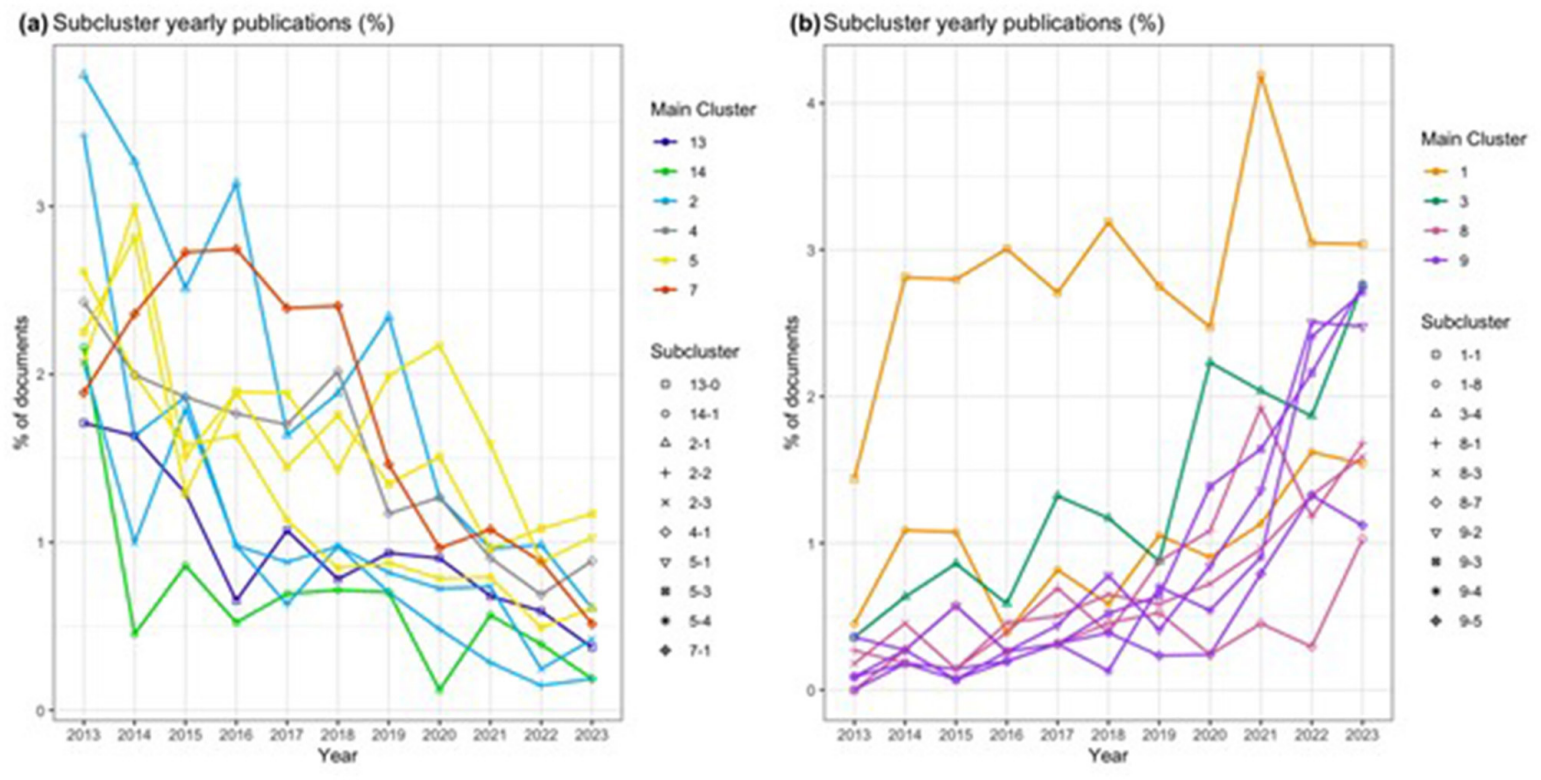
Patent research subclusters with the largest **(A)** decline and **(B)** growth over the past 10 years.

Larger subclusters, such as “Legal Frameworks and Challenges in Patent Systems” (2-1), “Factors Influencing Innovation and Technological Impact” (1-1), “Geographic Mobility and Knowledge Spillovers” (1-2), indicate areas of extensive research activity. The figure also reveals emerging trends, particularly in environmental sustainability and corporate innovation, as evidenced by the concentration of recent subclusters from clusters 8 and 9, all about sustainability and green innovation on the right side of the chart. An interesting case is that of “Traditional Chinese Medicines for Viral Infections” (12-1), which also appears as a recent subcluster due to an increase in publications related to alternative medicine and the IP challenges triggered due to the coronavirus disease 2019 (COVID-19) pandemic. The upper right quadrant of the chart showcases high-impact recent research, exemplified by subclusters “Patent Analytics in Drug Discovery and Network Pharmacology” (11-4) and “Patent Analytics in Energy Sectors” (3-4), which have quickly gained significant attention. Another aspect that characterizes research fronts is that of fast-growing research, as shown in Figure 5, depicting the subclusters with the largest decline and growth.

Subclusters related to research on University-Industry collaboration (Cluster 5) and Patent Systems and Biomedical Innovations (Cluster 2) are among the research trends with the larger decline. Growth is aligned with recency as, again, subclusters in the areas of sustainability and corporate innovation show rapid growth. Two subclusters from “Patent Analytics and Innovation Dynamics” also show fast growth even though they belong to the established cluster 1: Factors Influencing Innovation and Technological Impact (1-1), Patent Analytics in Regional and Technological Innovation (1-8), meaning that these topics have been and continue to be relevant for patent research. Subclusters with outstanding characteristics have been selected for further description in the following sections.

#### 4.2.1 Biggest subclusters

Factors Influencing Innovation and Technological Impact (1-1): This cluster explores how knowledge recombination, organizational learning structures, and search behaviors influence innovation effectiveness (Hausman et al., 1984; Rosenkopf and Nerkar, 2001). The research examines the roles of knowledge relationship intensity, neighboring knowledge concentration, technology sourcing strategies, and team-based research in generating high-impact patents (Wuchty et al., 2007).

Geographic Mobility and Knowledge Spillovers (1-2): The research in this cluster examines how geographic factors [e.g., geographical proximity (Jaffe et al., 1993), transportation infrastructure (Cao et al., 2024)], and inventor mobility influence the localization and dissemination of knowledge. It explores the role of alliances and labor networks in facilitating knowledge transfer across geographical and technological boundaries. Additionally, this cluster investigates spillover effects between different sectors [such as defense to civilian (Riebe et al., 2024)] and compares innovation patterns among different groups of inventors, including immigrants.

Legal Frameworks and Challenges in Patent Systems (2-1): This cluster covers the nuances of patent protection, examining discrepancies between copyright and patent standards, particularly in areas like design patents. For example, we find research investigating how patents function as both profit-making tools and mechanisms for technological foresight, using examples from specific sectors such as hydrogen energy (Erivantseva et al., 2024). It also addresses common pitfalls in patent protection and strategies for effective claim drafting (Merges and Nelson, 1990). The cluster encompasses discussions on the economic dynamics of patent scope, the fundamental principles and purposes of the patent system (Kitch, 1977), and the concept of rational ignorance within patent offices (Lemley, 2001). Additionally, it explores critiques of the current patent system, including movements advocating for open access to innovation.

#### 4.2.2 Highly cited subclusters

Strategic Alliance Governance and Innovation Outcomes (1-5): This cluster focuses on using patent analytics to understand innovation dynamics and strategic partnerships. It explores the application of advanced techniques like machine learning in patent analysis, the role of strategic alliances in technological innovation, and the relationship between various innovation indicators (Hagedoorn and Cloodt, 2003; Hanisch, 2024). The research in this cluster has evolved from early studies on patent statistics as innovation proxies to more sophisticated analyses of knowledge transfers and the impact of acquisitions on innovation performance.

Innovative Drug Development and Repurposing (7-7): This cluster traces the evolution of drug repurposing and innovative therapeutic development, emphasizing both natural products and existing drugs. The research trajectory reflects a shift from traditional drug discovery to more efficient, cost-effective strategies (Paul et al., 2010). Research in this cluster leans toward computational methods for drug repositioning and renewed interest in natural products as therapeutic sources (Malla et al., 2024; Singla et al., 2023). It emphasizes the critical role of patents in protecting new applications of repurposed drugs and natural compounds, addressing unmet medical needs, and improving drug development efficiency.

Patent Analytics in Drug Discovery and Network Pharmacology (11-4): This cluster tackles research from traditional database-driven approaches (Gaulton et al., 2017; Wang et al., 2020) to more sophisticated network pharmacology analyses and machine-learning applications (Xia et al., 2024; Zheng et al., 2024). This cluster emphasizes the growing importance of patent analytics in identifying new drug candidates and leveraging traditional medicine knowledge in modern pharmaceutical research.

#### 4.2.3 Recent subclusters

Impact of Policies and Corporate Factors on Innovation (8-3): This cluster examines how national policies, corporate structures, and cultural factors influence innovation outcomes, using patent data as a key metric. It explores the effects of initiatives like “Made in China 2025” (Chen K. J. et al., 2024) and US-China technology decoupling on firm performance and innovation (Han et al., 2024). The research investigates how corporate risk culture impacts innovation, particularly in innovative industries, and how different organizational forms (such as conglomerates and venture capital backing) affect R&D productivity. Studies in this cluster also analyze the dynamics of mergers and acquisitions in relation to patent portfolios and technological synergies (Bena and Li, 2014), highlighting how patent data can inform strategic corporate decisions.

Banking Financing R&D and Innovation (8-8): This research examines how the development of equity and credit markets, as well as banking deregulation, affects corporate patenting (Hsu et al., 2014). Studies in this cluster reveal that the ability to use patents as collateral [patent pledgeability (Dai et al., 2024)] positively impacts corporate patenting. The cluster also explores how financial constraints and debt financing influence innovation outcomes, using patent-based metrics to measure these effects (Shahzad et al., 2024).

Regional Dynamics of Green Technology Development (9-5): This cluster analyzes the role of digital governance and technological innovation in driving sustainable development, mainly in China, using patent analytics to assess the effects on natural resource management, energy efficiency, and urban green development (Chen K. et al., 2024; Lu and Li, 2024). Studies in this cluster also investigate the spatial and temporal distribution of environmental patents in China, assess the effectiveness of patent subsidy programs, and scrutinize regional disparities in innovation capabilities and eco-efficiency across Chinese cities. Although the focus is on China, regional studies from other countries are also present.

Patent Analytics in Carbon Reduction Technologies (9-3): This cluster explores the role of technological innovation in addressing environmental sustainability challenges, examining how patents influence green innovation and environmental degradation across various geographic contexts (Albino et al., 2014; Hashmi and Alam, 2019). For instance, studies analyze the impact of technological innovation on the ecological footprint of innovative countries, the effectiveness of China's green patent fast-track system (Xu A. T. et al., 2024), and the role of environmental-related patents in Nordic countries (Alola et al., 2024). The research also highlights development trends in low-carbon energy technologies and examines the dynamic interplay between innovation, environmental regulation, CO2 emissions, population, and economic growth in countries part of the Organization for Economic Co-operation and Development (OECD).

Drivers of Corporate Environmental Innovation (9-4): This cluster investigates the various factors driving green innovation and environmental sustainability, focusing on the impact of green finance, external resources, corporate Environmental, Social, and Governance (ESG) ratings, place-based policies, and institutional pressures on the development of green patents (Berrone et al., 2013; Cainelli et al., 2015). It also explores the impact of place-based policies, such as the revitalization of old revolutionary base areas in China (Nie et al., 2024), on urban green technology innovation and how institutional pressures drive environmental innovation in polluting industries.

#### 4.2.4 Rapidly growing subclusters

Patent Analytics in Regional and Technological Innovation (1-8): Studies in this cluster investigate drivers of regional diversification in industrial districts, the impact of technology flows through patent transactions on regional specialization (Liu et al., 2024), and the integration of AI into green technologies. The research also examines how related and unrelated technological variety influences innovation output at the city or state levels and how urbanization affects economic development and knowledge creation (Bettencourt et al., 2007; Castaldi et al., 2015).

Patent Analytics in Energy Sectors (3-4): Focusing on energy-related technologies, this cluster utilizes patent analytics to track innovations in hydrogen fuel cells, lithium-based batteries, CO2 capture, and redox flow batteries (Li et al., 2013; Zhou et al., 2024). It highlights the importance of collaborative networks and patent analysis in informing policy decisions and technological development strategies. Studies also examine patents related to CO2 capture technologies and the commercial development of all-vanadium redox flow batteries for energy storage (Kear et al., 2012).

Corporate Governance and Financial Factors in Firm Innovation (8-1): This cluster examines various factors affecting firm-level innovation, including corporate lobbying, institutional ownership, political alignment of executives, financial analyst pressure, and CEO overconfidence (Hirshleifer et al., 2012; Jiao and Lu, 2024). It uses patent metrics to measure innovation outcomes and explore the complex interplay of these factors. Studies investigate how corporate lobbying enhances firm innovation outcomes, the impact of institutional stock ownership on corporate innovation (Simeth and Wehrheim, 2024), and the influence of political partisanship among firm executives on innovation outcomes (Jiao et al., 2024).

Public Financing R&D and Innovation (8-7): The research in this cluster emphasizes the importance of addressing financial constraints and tailoring policy approaches to enhance R&D investments and patenting activities (Czarnitzki et al., 2007). Studies explore the role of financial technology in promoting regional innovation and the effectiveness of public R&D subsidies (Wang et al., 2024). The cluster also includes research on the funding gap in R&D (Hall, 2002), the effects of government-sponsored commercial R&D subsidies.

Environmental Regulation and Green Patent Quality (9-2): This cluster explores the relationship between environmental policies, pollution levels, and green innovation. It uses patent data to analyze the impact of carbon intensity policies, air pollution, and environmental regulations on technological innovation and regional carbon emission reduction (Brunnermeier and Cohen, 2003; Jaffe and Palmer, 1997). The cluster also includes research on the relationship between environmental compliance expenditures and R&D investment, global trends in environmental patenting, and the impact of pollution abatement expenditures on environmental innovation in manufacturing industries (Xu J. H. et al., 2024; Xu S. C. et al., 2024).

### 4.3 Knowledge cross-sharing

**Figure 6:**
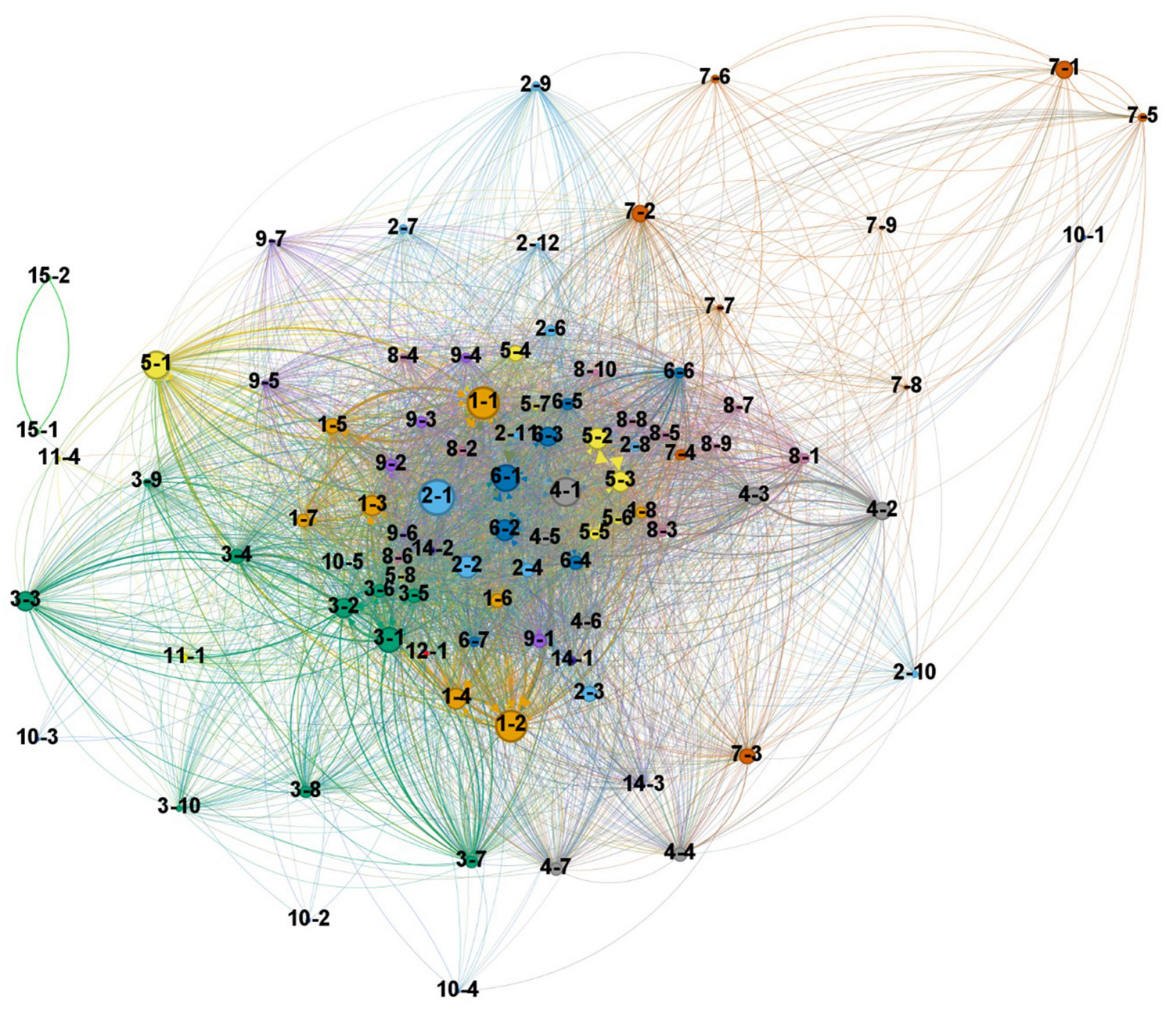
Network visualization of patent analytics research subclusters. Nodes represent individual subclusters, with node color indicating the main cluster to which each subcluster belongs. Node size reflects the size based on the number of documents. Edges represent aggregate citations between subclusters.

**Figure 7:**
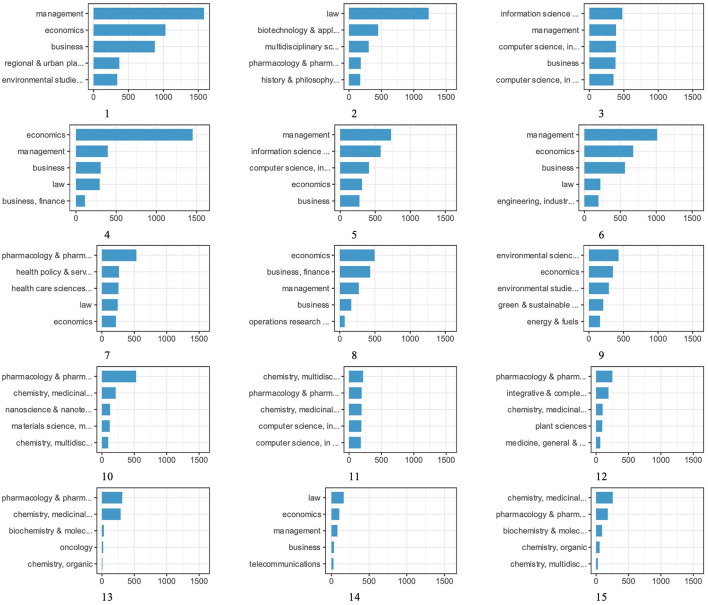
Most frequent 5 Web of Science categories by number of articles per cluster.

The citation network revealed clusters and subclusters representing focal topics of research. Academic articles were used as nodes in the initial network in Figure 2. An aggregation of the nodes at the subcluster level reveals the knowledge structure across the topics. Figure 6 presents a network visualization that captures the interactions and knowledge flows between different areas of patent analytics research as represented by the subclusters, being this a macro-level perspective on how various subclusters relate to and influence each other.

In this network, the central position of, for instance, subclusters 6-1 (Patent Value and Citations), and 6-2 (Patent Strategies and Innovation) indicates these are foundational areas in patent analytics research. Their centrality suggests they serve as active sources and sinks of insights that are widely applicable across the field. The network also reveals thematic coherence within the main research areas, as the subclusters tend to agglomerate near other subclusters in the same main topic (i.e., same cluster color). We observe that subclusters Advanced Methods in Patent Analytics and Technology Forecasting (Cluster 3) tend to interact more with each other, suggesting a well-integrated body, yet they share connections to clusters 1, 6, and 9, indicating their relevance for these other research areas.

The dense center of the network, dominated by subclusters from the main clusters 1, 2, 4, and 6, represents the core of patent research. The sparser periphery, including specialized topics like specific drug delivery systems or environmental technologies, represents more focused applications of patent analytics.

Another possible representation of the subcluster's relationship is through semantic analysis, for example, by measuring the text-similarity of the contents in each cluster. Such a perspective has been added in Figure A4. However, semantic analysis tends to place the subclusters of the same cluster near each other, and thus, it only has value as a confirmatory method. It confirms that the subcluster partitions from the citation network are also semantically coherent.

## 5 Discussion

Through a citation network analysis of over 27,000 academic articles, 15 main clusters and 93 subclusters were identified, revealing the landscape of patent research. The results show a steady increase in the use of patent documents in academic research. We note that the interest in academia is shared by two distinct but highly integrated groups, one that focuses on management and innovation studies and the other that is more applied to pharma and biomedical research. For instance, clusters such as “Patent Analytics and Innovation Dynamics” and “Advanced Methods in Patent Analytics and Technology Forecasting” dominate the first, while “Patent Systems and Biomedical Innovations” dominates the second. Emerging trends in environmental sustainability and biomedical innovations were identified, as evidenced by the recent and rapidly growing subclusters in these areas. The analysis also revealed the widespread integration of advanced analytical techniques, including AI and machine learning, across various domains of patent research. These findings provide a foundation for addressing the study's core research questions: how patent documents are used in academic research, current trends in patent research, and the role of patent analytics methods within the broader scope of patent research. The following discussion examines these questions in detail, drawing insights from the identified clusters and their interrelationships.

How is patent information used in academic research?

We found that patent bibliographic data and information are used extensively and diversely in academic research, serving as rich sources of information for understanding technological innovation, knowledge flows, and economic impacts. The cluster analysis revealed several key applications of patent data in research:

Indicators of innovation: Patents are widely used as proxies for measuring innovative activity across different technological fields and geographic regions. This being the most mature use of patents in Innovation Management, as evidenced by the seminal work of Soete (1979) and later consolidated by Narin (1994) highlighting the similarities between patent bibliometrics and scientific literature bibliometrics, particularly in assessing national and inventor productivity.

Knowledge spillover analysis: The network visualization of subclusters (Figure 6) highlighted the importance of patent citations in tracing knowledge flows as inferred from the central place of subclusters 1-2 and 6-1, both on patent citation flows. These build upon the seminal work of Jaffe et al. (1993), who used patent citations to demonstrate the geographic localization of knowledge spillovers. Our findings suggest that this approach remains relevant, with studies on geographical proximity and spillover still being conducted and what has changed is the sophistication of the methods or the access to new datasets.

Technological forecasting: Several subclusters focus on using patent data to identify emerging technologies and predict future trends. Some of these, like most of the subclusters in cluster 3, prime the development of methods from patent data, while other subclusters focus on the application of such methods to bring forward-looking views on the fields (e.g., research on patent analytics for energy, green energy, and nano technologies) This streams aligns with the tech mining approaches (Porter and Newman, 2009), now being extended by the incorporation of more advanced data analytics techniques.

Innovation quality assessment: This covers research using patents as a benchmark to assess firms or innovation portfolios. Although there is a prominent background in patent counts and citations, some research emphasizes the multidimensional nature of patent quality, challenging simplistic metrics, thus aligning more with the concept of patent quality (Higham et al., 2021). Our analysis supports this view, showing diverse approaches to evaluating patent impact across different technological domains (e.g., as in subclusters 1-1, 8-4).

Policy and economic analysis: Patents are used to assess the effectiveness of R&D funding strategies. We observed subclusters focused on how different funding sources (public vs. private) influence patenting outcomes across various sectors (Shelton and Leydesdorff, 2012) and also research areas focused on the interplay between industry, academia, and government trough different initiatives of polices (Ivanova et al., 2017).

Corporate strategy and competitive intelligence: Patent analysis is extensively used to understand competitive landscapes and inform strategic decision-making. Most of the clusters related to innovation management are geared toward this use case.

Interdisciplinary research: We observe the integration of patent analysis with diverse fields such as environmental sustainability, biomedical innovations, and digital technologies, showcasing the versatility of patent data in being a supporting data source for multiple disciplines.

What are the current trends in patent research?

Current trends in patent research reflect a shift toward more sophisticated, context-aware approaches in patent analysis. This evolution addresses Meyer's (2000) call for recognizing the unique characteristics of patent citations compared to scientific citations. The emergence of tech mining and AI-driven analytics in patent research, as highlighted in recent and growing subclusters, suggests a shift toward more data-intensive and nuanced approaches to extracting value from patent documents.

A prominent trend is the integration of environmental sustainability and green innovation with patent analytics. Subclusters focusing on “Patent Analytics in Carbon Reduction Technologies” (9-3) and “Drivers of Corporate Environmental Innovation” (9-4) have shown recent emergence and significant growth. This trend reflects the broader societal focus on sustainable development and demonstrates how patent analysis is being applied to track and foster eco-friendly innovations.

The rise of micro-level scientometrics, focusing on detailed interactions within organizations and among individuals, represents another current trend. This approach, highlighted in Zhang et al. (2017) work on scientometrics for tech mining, is evident in growing subclusters that examine the impact of corporate structures, funding sources, and individual inventor characteristics on innovation outcomes.

Another notable trend is the application of advanced data analytics and artificial intelligence in patent research. Subcluster 3-4, “Patent Analytics in Energy Sectors,” exemplifies this trend, showcasing the use of sophisticated analytical techniques to understand technological evolution in energy-related fields. While this subcluster is more on the applied side, it is embedded within cluster 3 of advanced methods, suggesting a need in the field of energy innovation to leverage the most up-to-date methodologies for innovation analysis. The integration of patent analytics into unrelated but specific research has been long foreseen as part of the discussions on tech mining (Porter and Newman, 2009).

Lastly, we observe a growing trend in analyzing the intersection of patent data with other data sources, such as scientific publications, market data, and policy information. This holistic approach to innovation analysis, evident in subclusters like 1-5 “Strategic Alliance Governance and Innovation Outcomes,” represents an evolution from earlier, more siloed approaches to integrated patent analysis. Advances in natural language processing facilitate the integration of diverse data sources.

Role of patent analytics methods within the larger scope of patent research?

The role of patent analytics methods within the larger scope of patent research has evolved to become increasingly central and sophisticated. Patent analytics methods serve as critical tools for identifying and quantifying technological emergence (Carley et al., 2018). Growing subclusters focused on advanced methods in patent analytics indicate a shift toward more data-driven and objective approaches to understanding innovation trajectories.

These methods facilitate the integration of diverse data sources, allowing for a more comprehensive understanding of innovation ecosystems. There is a trend in patent analytics methods now routinely combining patent data with scientific publications (Mejia and Kajikawa, 2020), market information, and even social media (Orduna-Malea and Font-Julian, 2022) data to provide richer insights.

Patent analytics methods play a crucial role in enhancing the strategic value of patent research for both policymakers and industry practitioners. The growth of subclusters focused on competitive intelligence and strategic decision-making underscores how these methods are bridging the gap between academic research and practical applications.

### 5.1 Patent analytics: a framework

The citation network analysis revealed the “organic” structure and evolution of topics based on authors' research preferences and citation patterns. This method allowed us to process a large volume of papers and identify focal topics in the research landscape. However, to enhance the practical utility of these insights, we conducted a deeper analysis of the subcluster contents and meanings. Building upon this systematic, computer-assisted analysis of citation networks in patent research, we propose a conceptual framework that synthesizes and organizes the field into five core components. Table 1 shows the proposed framework, which consists of five core components and the corresponding subclusters in our analysis. The core components are:

(1) Fundamentals of patents systems
(2) Patents as indicators
(3) Methodological development of patent analytics
(4) IP management practice
(5) Patent analytics applications

Patent research has come a long way since the early review attempts of the field (e.g., Basberg, 1987). Now, basis analytics like patent counts and citations, regional innovation benchmarking, and patent relevance assessment are just a small (yet important) part of the big picture the field has become. By organizing the subclusters into these components, we aim to provide a clearer picture of how different and cutting-edge research streams contribute to the overall understanding and practice of patent analytics. As can be seen in Table 2, the framework is organized around fundamental aspects of patent analytics research and practice. This approach allows us to highlight how different streams of research contribute to explaining various aspects of the patent analytics process and its applications. The framework can provide a practical and actionable structure for researchers and practitioners in the field of patent analytics. It will also work as a guidance for authors, reviewers, and editors in Patent Analytics section of the journal.

**Table 2:** List of subclusters in the core components of patent research and analytics.

**Id**	**Core components and subclusters**
(1) Fundamentals of patents systems: *Research on the legal, economic, and policy aspects of patent systems. It includes studies on patent laws, regulations, ethics, histories, and policies, as well as their impacts on innovation and economic growth. The research objective of this foundational core is to provide the context for understanding how patent systems function and evolve and also to provide evidence for the design and legitimation of patent systems*.	
2-1	Legal frameworks and challenges in patent systems
2-2	Patenting genetic data and human biological materials
2-3	Neurotechnology and cell therapy patent regulation
2-5	IP challenges in biomedical patents
2-6	Socio-cultural discussions of patents and patent systems
2-10	Patent policies and pharmaceutical innovation for vulnerable groups
2-12	Patent analytics in corporate power and policy dynamics
4-1	Impact of patent policies on innovation and technology spillover
4-2	Global patent protection analysis and determinants
4-3	Determinants of innovation and patent activity across different economies
4-5	Patent policy and economic growth
4-6	Regional patent competition and technology diffusion
4-7	Historical and sectoral analysis of patents and innovation
7-1	Biosimilars development and regulation
7-2	Pharmaceutical patents and market exclusivity
7-3	Patent policies and access to medicines in developing countries
7-4	Pharmaceutical price dynamics and market entry
9-1	Environmental policy impact on green patents
14-2	Impact of technology standards on patenting and innovation
(2) Patents as indicators: *This component focuses on the use of patent data as indicators of innovation, technological progress, and economic performance. It includes research on patent citations, knowledge spillovers, and the relationship between patents and various economic and social factors. It also includes quantitative and monetary valuation of patents and analysis of economic and social outcomes. The main research objective of this core is to understand innovation processes, including technological, business, and economic development, rather than patent and patent systems*.	
1-1	Factors influencing innovation and technological impact
1-2	Geographic mobility and knowledge spillovers
1-3	Demographic-driven regional innovation
1-4	Structural analysis of innovation networks
1-6	Patent analytics in multinational corporations
1-7	R&D investment impact on firm innovation efficiency
1-8	Patent analytics in regional and technological innovation
5-1	Interplay between scientific research and technological innovation in patent analytics
5-2	Efficiency and dynamics of university-industry collaboration and technology transfer
5-3	University patenting and commercialization
5-5	Impact of university research on patent landscapes
5-6	Gender disparities in patenting
6-1	Patent value and citations
6-3	Role of patents in innovation and economic performance
7-9	Quality, safety, and market dynamics of generic and off-patent pharmaceuticals
8-10	External influences on innovation and patenting activities
14-3	Role of patents and technological innovation in economic growth and trade performance
(3) Methodological development of patent analytics: *Development and refinement of methods and tools for analyzing patent data. The research objective of this core is to provide methodology for searching patents, illustrating patent landscape, describing patent trends, analyzing technological development, and identifying technological and business opportunities. The methods include text mining, machine learning, and other advanced analytical techniques applied to patents and databases*.	
3-1	Patent analytics for technological trends and innovation assessment
3-2	Text Mining and machine learning in patent analytics
3-3	Data-driven approaches in patent analytics
3-5	Patent citation networks and development pathway analysis
3-6	NLP-based patent mining for innovation gaps
3-7	Patent analytics and technology convergence
3-8	Patent-driven product design and knowledge transfer
11-1	Drug design and patent analytics
11-2	Chemical patent information retrieval
11-3	Patent search strategies
11-4	Patent analytics in drug discovery and network pharmacology
(4) IP management practice: *This component focuses on practical aspects of managing intellectual property, particularly patents, within organizations. It includes research on R&D strategies, patent strategies, portfolio management, licensing, financing, and the integration of patent analytics into business decision-making processes. Case studies in the medical and healthcare sectors are active in this core, which reflects the strong impacts of patenting in the sectors. The research objective of this core is to derive practical implications based on existing academic expertise and to provide feedback to academic expertise based on practical cases*.	
1-5	Strategic alliance governance and innovation outcomes
2-4	Intellectual property strategies in healthcare innovation
5-8	Impact of knowledge disclosure and intellectual property strategies on firm innovation and performance
6-2	Patent strategies and innovation
6-4	Strategic patent commercialization and licensing dynamics
6-5	Venture capital and patent-based innovation financing
6-6	Empirical analysis and trends in patent licensing and innovation
6-7	Impact of intellectual property analytics on innovation and economic performance
8-1	Corporate governance and financial factors in firm innovation
8-2	The role of R&D and patents in economic performance and technology acquisition
8-3	Impact of policies and corporate factors on innovation
8-4	Innovation efficiency and benchmarking
8-5	Organizational and environmental factors in corporate innovation
8-6	Impact of patent analytics and R&D on firm innovation and financial performance
8-7	Public financing R&D and innovation
8-8	Banking financing R&D and innovation
8-9	Patent analytics in innovation and economic policy
14-1	Evolving dynamics of SEP licensing and litigation
(5) Patent analytics applications: *Application of patent analytics in specific fields or industries. The research objective of this core is to apply existing analytical methods in each technological topic and to gain insights for driving technological development and innovation in various sectors, from pharmaceuticals to green technologies*.	
2-7	Patent analytics in natural and genetic resources
2-8	Patent analytics in agricultural biotechnology
2-9	CRISPR and precision agriculture patents
2-11	Patent analytics in life sciences innovation
3-4	Patent analytics in energy sectors
3-9	Sector-specific applications of patent analytics in innovation
3-10	Patent analysis in environmental and health sciences
4-4	Pharmaceutical patents and global healthcare access
5-4	Patent analytics in nanotechnology
5-7	Industry-specific innovation networks
7-5	Patent challenges and opportunities in biosimilars
7-6	Drug pricing policy analysis
7-7	Innovative drug development and repurposing
7-8	Patents and access to diabetes medications
9-2	Environmental regulation and green patent quality
9-3	Patent analytics in carbon reduction technologies
9-4	Drivers of corporate environmental innovation
9-5	Regional dynamics of green technology development
9-6	Green Innovation systems and economic transformation
9-7	Impact of patenting activities on employment and innovation dynamics
10-1	Pharmaceutical formulations and solubility enhancement
10-2	Patent analytics in biomedical innovations and drug delivery systems
10-3	Innovative drug delivery systems and patent analytics
10-4	Nanoparticle drug delivery systems
10-5	Nanocarrier-based drug delivery systems
12-1	Traditional Chinese medicines for viral infections
15-1	Development and applications of carbonic anhydrase inhibitors
15-2	Patent landscape of carbonic anhydrase inhibitors

### 5.2 Future research directions

Despite the comprehensive nature of this study, there are limitations that present opportunities for future research. In the area of patent systems fundamentals, future research could explore the evolving nature of patent systems in the digital age, such as the potential integration of blockchain technology for improved transparency and efficiency. Studies on the impact of harmonization efforts in global patent systems, particularly in emerging economies, are needed. Research on the effectiveness of patent policies in promoting innovation in specific sectors, such as green technologies or artificial intelligence, could provide valuable insights.

Regarding patents as indicators, developing more sophisticated indicators that combine patent data with other data sources could provide a more holistic view of innovation dynamics. Future research could explore the use of patents as indicators of technological convergence and the potential for using patent indicators to predict emerging technologies or market trends, leveraging machine learning techniques.

In methodological development, advancements in natural language processing and machine learning offer exciting possibilities for patent analytics. Future research could focus on developing more accurate, efficient, and effective text mining techniques for patent documents, improving visualization techniques for large-scale patent data, and developing methodologies for real-time patent analytics.

Cross-cutting themes for future research include exploring how AI can enhance various aspects of patent research, examining how patent systems and analytics can promote sustainable innovation, studying how patent analytics can inform global innovation strategies, researching ethical implications and responsible use of patent analytics tools, developing tools and methodologies that make patent analytics more accessible to smaller organizations and individual inventors, exploring how patent data can be effectively combined with other data sources for more comprehensive innovation analysis, and conducting more sector-specific studies in rapidly evolving fields.

## 6 Conclusion

This study provided an overview of the current state and future directions of patent analysis in academic research. We identified 93 research streams from academic literature that use the patent document in any form; these topics were evaluated in terms of size, recency, citation impact, and growth, revealing relevant trends. These include an increased focus on AI methods and the application of patent analytics for sustainability and evaluation of corporate performance. We further organized the topics to reach a five-core component framework encompassing fundamentals of patent systems, patents as indicators, methodological developments, IP management practices, and applications. By proposing an integrated framework and identifying key trends and challenges, we contribute to both the theoretical understanding and practical application of patent analytics.

As patent data becomes increasingly accessible and analytical techniques more sophisticated, the field of patent analysis is poised to play an even more crucial role in informing innovation policy, guiding corporate strategy, and advancing our understanding of technological progress.

The evolution of patent analysis from simple citation counts to complex, AI-driven analyses reflects the growing recognition of patents as rich sources of technological and economic information. However, this evolution also brings new challenges in terms of data interpretation and methodological rigor. Future research should focus on addressing these challenges while continuing to explore novel applications of patent analytics across various domains of science and technology and also in various sectors, such as academia, business, and policy, to empower innovation.

## Data Availability

The original contributions presented in the study are included in the article/Supplementary material, further inquiries can be directed to the corresponding author.

## Appendix

The most frequent Web of Science categories show correspondence to the clusters' topical focus with dominance of Management, Law, and Economics for the largest clusters as shown in Figure 7. Pharma and Chemistry fields have also strong presence due to prevalence of journals on patent reviews in these fields. Cluster 3′s distinctive focus on patent analytics and forecasting is led by publications in Information Science. The most frequent categories per subcluster are shown in the Table A2.
