# Supplementary material for: Patent research in academic literature. Landscape and trends with a focus on patent analytics
Source: Front Res Metr Anal. 2025 Jan 8;9:1484685. doi: 10.3389/frma.2024.1484685 (PMC11751822; doi:10.3389/frma.2024.1484685)
Supplement: Supplementary file 1 [file Data_Sheet_1.docx]

Supplementary Material

# Supplementary Figures

***Figure A1.*** a) yearly publication trend; b) Top 20 journals by article count; c) Top 20 Web of Science categories by article count.


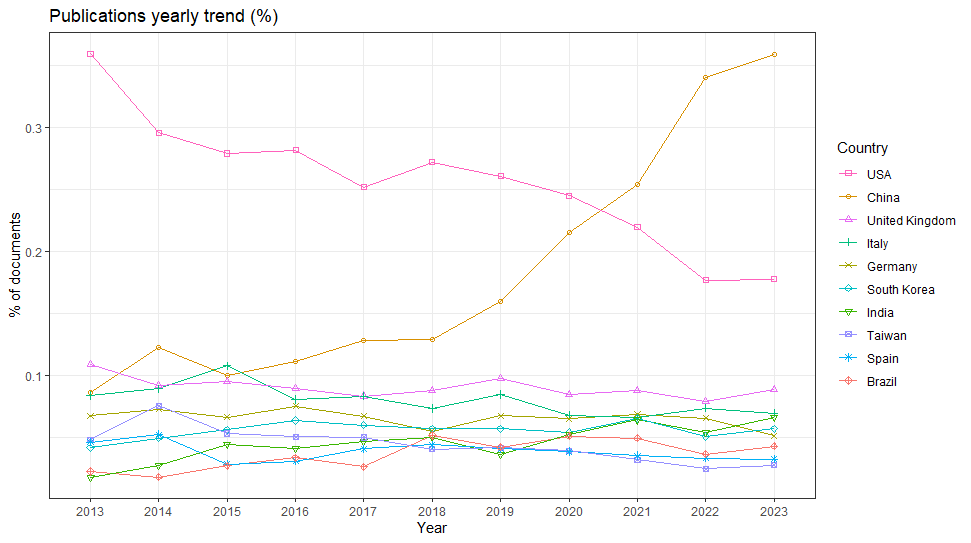


***Figure A2.*** Yearly publication trends of the ten most engaged countries by publication count over the past 20 years.


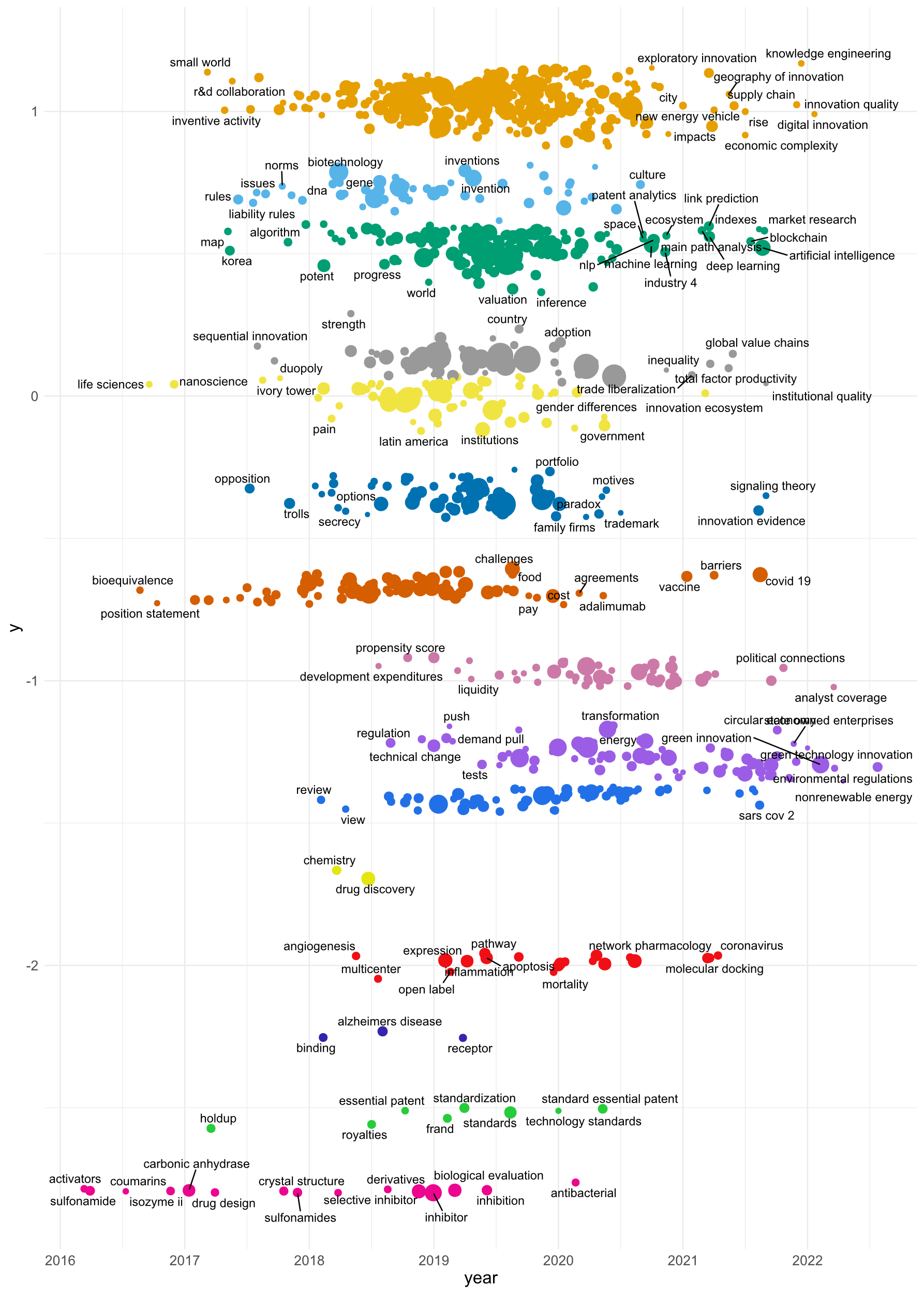


***Figure A3.*** Co-keyword analysis of the most frequent 1000 terms by publication count in the title, abstract, or author keyword. The figure represents the nodes of a co-keyword network without the edges. The nodes are plotted based on the average publication year over the x-axis and stacked based on the main clusters in which they occur the most. The y-axis shows the relative proximity of the term within each cluster.


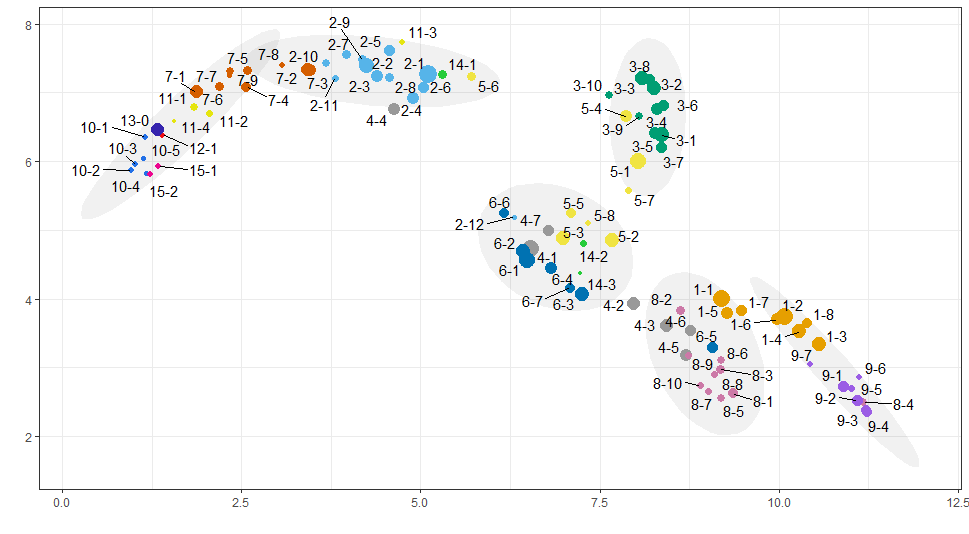


***Figure A4.*** Semantic map of patent analytics research subclusters. Each point represents a subcluster, with colors indicating main cluster affiliations and size reflecting the number of documents within each subcluster. The map is generated using UMAP dimensionality reduction of cosine similarity between subcluster text embeddings. The spatial arrangement illustrates thematic relationships, with six distinct groupings emerging: (1) Regional and Environmental Innovation Analytics, (2) Biomedical and Agricultural Patent Landscapes, (3) Strategic Patent Management and Economic Impact, (4) Innovation Drivers and Policy Influences, (5) Pharmaceutical Innovation and Drug Delivery Systems, and (6) Advanced Methods in Technological Innovation Analytics. The distribution and clustering of points reveal core research areas, specialized domains, and interdisciplinary connections within the field of patent analytics.

# Supplementary Tables

***Table A1.*** Subclusters. a. articles, b. ave. publication year, c. ave. citations, d. framework component*

| ID | Subcluster name | a | b | c | d |
| --- | --- | --- | --- | --- | --- |
| 1-1 | Factors Influencing Innovation and Technological Impact | 753 | 2,016.2 | 42.2 | 2 |
| 1-2 | Geographic Mobility and Knowledge Spillovers | 732 | 2,015.9 | 32.9 | 2 |
| 1-3 | Demographic-Driven Regional Innovation | 461 | 2,013.8 | 29.3 | 2 |
| 1-4 | Structural Analysis of Innovation Networks | 455 | 2,017.5 | 30.4 | 2 |
| 1-5 | Strategic Alliance Governance and Innovation Outcomes | 324 | 2,014.1 | 52.4 | 4 |
| 1-6 | Patent Analytics in Multinational Corporations | 296 | 2,014.0 | 32.7 | 2 |
| 1-7 | R&D Investment Impact on Firm Innovation Efficiency | 264 | 2,013.5 | 27.7 | 2 |
| 1-8 | Patent Analytics in Regional and Technological Innovation | 222 | 2,017.9 | 18.3 | 2 |
| 2-1 | Legal Frameworks and Challenges in Patent Systems | 831 | 2,009.5 | 22.0 | 1 |
| 2-2 | Patenting Genetic Data and Human Biological Materials | 504 | 2,009.5 | 11.0 | 1 |
| 2-3 | Neurotechnology and Cell Therapy Patent Regulation | 322 | 2,011.7 | 7.5 | 1 |
| 2-4 | Intellectual Property Strategies in Healthcare Innovation | 286 | 2,011.8 | 17.8 | 4 |
| 2-5 | IP challenges in biomedical patents | 240 | 2,000.0 | 5.7 | 1 |
| 2-6 | Socio-cultural discussions of patents and patent systems | 229 | 2,007.8 | 8.7 | 1 |
| 2-7 | Patent Analytics in Natural and Genetic Resources | 163 | 2,012.1 | 15.4 | 5 |
| 2-8 | Patent Analytics in Agricultural Biotechnology | 162 | 2,004.5 | 10.6 | 5 |
| 2-9 | CRISPR and Precision Agriculture Patents | 155 | 2,016.7 | 12.1 | 5 |
| 2-10 | Patent Policies and Pharmaceutical Innovation for Vulnerable Groups | 114 | 2,005.5 | 10.3 | 1 |
| 2-11 | Patent Analytics in Life Sciences Innovation | 105 | 2,017.2 | 6.2 | 5 |
| 2-12 | Patent Analytics in Corporate Power and Policy Dynamics | 56 | 2,012.6 | 14.7 | 1 |
| 3-1 | Patent Analytics for Technological Trends and Innovation Assessment | 561 | 2,015.9 | 14.2 | 3 |
| 3-2 | Text Mining and Machine Learning in Patent Analytics | 437 | 2,016.5 | 12.4 | 3 |
| 3-3 | Data-Driven Approaches in Patent Analytics | 429 | 2,016.6 | 7.9 | 3 |
| 3-4 | Patent Analytics in Energy Sectors | 302 | 2,019.4 | 18.4 | 5 |
| 3-5 | Patent Citation Networks and Development Pathway Analysis | 295 | 2,017.3 | 13.5 | 3 |
| 3-6 | NLP-Based Patent Mining for Innovation Gaps | 260 | 2,017.2 | 13.6 | 3 |
| 3-7 | Patent Analytics and Technology Convergence | 260 | 2,017.1 | 11.8 | 3 |
| 3-8 | Patent-Driven Product Design and Knowledge Transfer | 246 | 2,016.1 | 12.6 | 3 |
| 3-9 | Sector-Specific Applications of Patent Analytics in Innovation | 134 | 2,017.7 | 8.2 | 5 |
| 3-10 | Patent Analysis in Environmental and Health Sciences | 106 | 2,018.5 | 13.5 | 5 |
| 4-1 | Impact of Patent Policies on Innovation and Technology Spillover | 674 | 2,009.2 | 20.2 | 1 |
| 4-2 | Global Patent Protection Analysis and Determinants | 386 | 2,013.5 | 18.3 | 1 |
| 4-3 | Determinants of Innovation and Patent Activity Across Different Economies | 381 | 2,016.0 | 18.6 | 1 |
| 4-4 | Pharmaceutical Patents and Global Healthcare Access | 295 | 2,012.7 | 12.5 | 5 |
| 4-5 | Patent Policy and Economic Growth | 285 | 2,014.8 | 25.1 | 1 |
| 4-6 | Regional Patent Competition and Technology Diffusion | 259 | 2,016.2 | 17.1 | 1 |
| 4-7 | Historical and Sectoral Analysis of Patents and Innovation | 256 | 2,009.5 | 11.0 | 1 |
| 5-1 | Interplay between Scientific Research and Technological Innovation in Patent Analytics | 625 | 2,010.8 | 18.6 | 2 |
| 5-2 | Efficiency and Dynamics of University-Industry Collaboration and Technology Transfer | 481 | 2,014.8 | 33.4 | 2 |
| 5-3 | University Patenting and Commercialization | 449 | 2,013.1 | 22.1 | 2 |
| 5-4 | Patent Analytics in Nanotechnology | 339 | 2,013.6 | 21.4 | 5 |
| 5-5 | Impact of University Research on Patent Landscapes | 218 | 2,013.4 | 25.8 | 2 |
| 5-6 | Gender Disparities in Patenting | 141 | 2,017.5 | 9.5 | 2 |
| 5-7 | Industry-Specific Innovation Networks | 100 | 2,015.0 | 13.0 | 5 |
| 5-8 | Impact of Knowledge Disclosure and Intellectual Property Strategies on Firm Innovation and Performance | 77 | 2,015.1 | 16.9 | 4 |
| 6-1 | Patent Value and Citations | 599 | 2,015.4 | 22.6 | 2 |
| 6-2 | Patent Strategies and Innovation | 454 | 2,014.7 | 18.3 | 4 |
| 6-3 | Role of Patents in Innovation and Economic Performance | 422 | 2,014.6 | 21.3 | 2 |
| 6-4 | Strategic Patent Commercialization and Licensing Dynamics | 299 | 2,015.5 | 18.6 | 4 |
| 6-5 | Venture Capital and Patent-Based Innovation Financing | 235 | 2,017.6 | 18.3 | 4 |
| 6-6 | Empirical Analysis and Trends in Patent Licensing and Innovation | 218 | 2,017.9 | 8.9 | 4 |
| 6-7 | Impact of Intellectual Property Analytics on Innovation and Economic Performance | 187 | 2,014.9 | 18.8 | 4 |
| 7-1 | Biosimilars Development and Regulation | 376 | 2,015.7 | 18.8 | 1 |
| 7-2 | Pharmaceutical Patents and Market Exclusivity | 361 | 2,016.5 | 11.6 | 1 |
| 7-3 | Patent Policies and Access to Medicines in Developing Countries | 340 | 2,014.7 | 10.2 | 1 |
| 7-4 | Pharmaceutical Price Dynamics and Market Entry | 210 | 2,011.7 | 17.4 | 1 |
| 7-5 | Patent Challenges and Opportunities in Biosimilars | 166 | 2,017.7 | 7.7 | 5 |
| 7-6 | Drug Pricing Policy Analysis | 148 | 2,016.5 | 15.8 | 5 |
| 7-7 | Innovative Drug Development and Repurposing | 111 | 2,016.0 | 34.9 | 5 |
| 7-8 | Patents and Access to Diabetes Medications | 72 | 2,019.3 | 4.2 | 5 |
| 7-9 | Quality, Safety, and Market Dynamics of Generic and Off-Patent Pharmaceuticals | 60 | 2,012.6 | 17.0 | 2 |
| 8-1 | Corporate Governance and Financial Factors in Firm Innovation | 188 | 2,020.3 | 17.4 | 4 |
| 8-2 | The Role of R&D and Patents in Economic Performance and Technology Acquisition | 156 | 2,009.3 | 23.2 | 4 |
| 8-3 | Impact of Policies and Corporate Factors on Innovation | 151 | 2,020.5 | 15.6 | 4 |
| 8-4 | Innovation Efficiency and Benchmarking | 129 | 2,015.2 | 20.9 | 4 |
| 8-5 | Organizational and Environmental Factors in Corporate Innovation | 124 | 2,018.3 | 21.8 | 4 |
| 8-6 | Impact of Patent Analytics and R&D on Firm Innovation and Financial Performance | 115 | 2,015.5 | 36.8 | 4 |
| 8-7 | Public Financing R&D and Innovation | 99 | 2,017.2 | 29.4 | 4 |
| 8-8 | Banking Financing R&D and Innovation | 96 | 2,020.5 | 14.3 | 4 |
| 8-9 | Patent Analytics in Innovation and Economic Policy | 86 | 2,020.0 | 10.0 | 4 |
| 8-10 | External Influences on Innovation and Patenting Activities | 85 | 2,017.5 | 35.0 | 2 |
| 9-1 | Environmental Policy Impact on Green Patents | 269 | 2,017.4 | 26.1 | 1 |
| 9-2 | Environmental Regulation and Green Patent Quality | 241 | 2,019.1 | 21.9 | 5 |
| 9-3 | Patent Analytics in Carbon Reduction Technologies | 217 | 2,021.3 | 10.1 | 5 |
| 9-4 | Drivers of Corporate Environmental Innovation | 189 | 2,021.6 | 11.4 | 5 |
| 9-5 | Regional Dynamics of Green Technology Development | 101 | 2,020.7 | 8.1 | 5 |
| 9-6 | Green Innovation Systems and Economic Transformation | 72 | 2,020.1 | 12.9 | 5 |
| 9-7 | Impact of Patenting Activities on Employment and Innovation Dynamics | 69 | 2,017.5 | 12.3 | 5 |
| 10-1 | Pharmaceutical Formulations and Solubility Enhancement | 78 | 2,014.8 | 36.1 | 5 |
| 10-2 | Patent Analytics in Biomedical Innovations and Drug Delivery Systems | 70 | 2,018.2 | 57.7 | 5 |
| 10-3 | Innovative Drug Delivery Systems and Patent Analytics | 66 | 2,020.2 | 17.8 | 5 |
| 10-4 | Nanoparticle Drug Delivery Systems | 61 | 2,019.7 | 19.6 | 5 |
| 10-5 | Nanocarrier-Based Drug Delivery Systems | 55 | 2,017.8 | 52.2 | 5 |
| 11-1 | Drug Design and Patent Analytics | 137 | 2,015.6 | 29.3 | 3 |
| 11-2 | Chemical Patent Information Retrieval | 86 | 1,996.3 | 13.9 | 3 |
| 11-3 | Patent Search Strategies | 69 | 1,983.2 | 3.0 | 3 |
| 11-4 | Patent Analytics in Drug Discovery and Network Pharmacology | 53 | 2,020.1 | 48.4 | 3 |
| 12-1 | Traditional Chineses Medicines for Viral Infections | 69 | 2,020.4 | 10.0 | 5 |
| 14-1 | Evolving Dynamics of SEP Licensing and Litigation | 148 | 2,014.7 | 9.0 | 4 |
| 14-2 | Impact of Technology Standards on Patenting and Innovation | 93 | 2,016.4 | 12.2 | 1 |
| 14-3 | Role of Patents and Technological Innovation in Economic Growth and Trade Performance | 54 | 2,015.9 | 7.0 | 2 |
| 15-1 | Development and Applications of Carbonic Anhydrase Inhibitors | 80 | 2,015.4 | 31.9 | 5 |
| 15-2 | Patent Landscape of Carbonic Anhydrase Inhibitors | 80 | 2,017.5 | 33.6 | 5 |

***Table A2.*** Subclusters and the top 5 Web of Science categories by number of articles.

| ID | Web of Science Categories |
| --- | --- |
| 1-1 | management; business; economics; operations research & management science; engineering, industrial |
| 1-2 | economics; management; business; geography; environmental studies |
| 1-3 | economics; regional & urban planning; geography; management; environmental studies |
| 1-4 | management; economics; business; environmental studies; regional & urban planning |
| 1-5 | management; business; engineering, industrial; economics; operations research & management science |
| 1-6 | management; business; economics; operations research & management science; engineering, industrial |
| 1-7 | management; business; economics; engineering, industrial; operations research & management science |
| 1-8 | economics; geography; regional & urban planning; management; environmental studies |
| 2-1 | law; economics; business; management; biotechnology & applied microbiology |
| 2-2 | law; genetics & heredity; biotechnology & applied microbiology; multidisciplinary sciences; ethics |
| 2-3 | law; biotechnology & applied microbiology; ethics; cell & tissue engineering; multidisciplinary sciences |
| 2-4 | law; biotechnology & applied microbiology; multidisciplinary sciences; economics; management |
| 2-5 | multidisciplinary sciences; biotechnology & applied microbiology; law; genetics & heredity; history & philosophy of science |
| 2-6 | history & philosophy of science; law; multidisciplinary sciences; business; economics |
| 2-7 | pharmacology & pharmacy; law; chemistry, medicinal; biotechnology & applied microbiology; integrative & complementary medicine |
| 2-8 | biotechnology & applied microbiology; plant sciences; agronomy; law; multidisciplinary sciences |
| 2-9 | biotechnology & applied microbiology; law; multidisciplinary sciences; genetics & heredity; medical ethics |
| 2-10 | pharmacology & pharmacy; multidisciplinary sciences; chemistry, medicinal; pediatrics; biotechnology & applied microbiology |
| 2-11 | biotechnology & applied microbiology; immunology; law; pharmacology & pharmacy; ethics |
| 2-12 | law; economics; multidisciplinary sciences; political science; international relations |
| 3-1 | business; management; information science & library science; regional & urban planning; operations research & management science |
| 3-2 | engineering, electrical & electronic; information science & library science; computer science, interdisciplinary applications; computer science, artificial intelligence; business |
| 3-3 | computer science, information systems; computer science, artificial intelligence; information science & library science; computer science, theory & methods; computer science, interdisciplinary applications |
| 3-4 | energy & fuels; environmental sciences; green & sustainable science & technology; engineering, environmental; electrochemistry |
| 3-5 | information science & library science; management; computer science, interdisciplinary applications; business; multidisciplinary sciences |
| 3-6 | business; management; regional & urban planning; engineering, industrial; information science & library science |
| 3-7 | management; information science & library science; computer science, interdisciplinary applications; business; environmental sciences |
| 3-8 | engineering, manufacturing; engineering, multidisciplinary; engineering, industrial; engineering, mechanical; computer science, artificial intelligence |
| 3-9 | environmental sciences; green & sustainable science & technology; environmental studies; engineering, multidisciplinary; engineering, industrial |
| 3-10 | information science & library science; pharmacology & pharmacy; biotechnology & applied microbiology; chemistry, medicinal; chemistry, multidisciplinary |
| 4-1 | economics; management; law; business; operations research & management science |
| 4-2 | economics; business; management; international relations; business, finance |
| 4-3 | economics; management; business; law; regional & urban planning |
| 4-4 | economics; law; business; health policy & services; management |
| 4-5 | economics; management; business; business, finance; social sciences, mathematical methods |
| 4-6 | economics; management; business; environmental studies; green & sustainable science & technology |
| 4-7 | economics; history of social sciences; history; law; history & philosophy of science |
| 5-1 | information science & library science; computer science, interdisciplinary applications; management; computer science, information systems; economics |
| 5-2 | management; engineering, industrial; business; economics; education & educational research |
| 5-3 | management; economics; information science & library science; business; engineering, industrial |
| 5-4 | nanoscience & nanotechnology; materials science, multidisciplinary; information science & library science; management; chemistry, multidisciplinary |
| 5-5 | management; economics; information science & library science; computer science, interdisciplinary applications; business |
| 5-6 | multidisciplinary sciences; law; business; information science & library science; economics |
| 5-7 | information science & library science; computer science, interdisciplinary applications; management; business; economics |
| 5-8 | management; economics; business; information science & library science; computer science, interdisciplinary applications |
| 6-1 | management; economics; business; information science & library science; computer science, interdisciplinary applications |
| 6-2 | management; business; economics; law; operations research & management science |
| 6-3 | management; business; economics; engineering, industrial; operations research & management science |
| 6-4 | management; economics; business; engineering, industrial; law |
| 6-5 | management; business; economics; business, finance; engineering, industrial |
| 6-6 | economics; management; law; business; information science & library science |
| 6-7 | management; economics; business; business, finance; regional & urban planning |
| 7-1 | pharmacology & pharmacy; oncology; biotechnology & applied microbiology; medicine, research & experimental; chemistry, analytical |
| 7-2 | law; pharmacology & pharmacy; health policy & services; health care sciences & services; economics |
| 7-3 | law; public, environmental & occupational health; pharmacology & pharmacy; health policy & services; ethics |
| 7-4 | economics; health policy & services; health care sciences & services; pharmacology & pharmacy; management |
| 7-5 | pharmacology & pharmacy; health care sciences & services; health policy & services; medicine, research & experimental; biotechnology & applied microbiology |
| 7-6 | pharmacology & pharmacy; health care sciences & services; health policy & services; medicine, general & internal; public, environmental & occupational health |
| 7-7 | pharmacology & pharmacy; chemistry, medicinal; law; oncology; medicine, research & experimental |
| 7-8 | medicine, general & internal; biotechnology & applied microbiology; law; pharmacology & pharmacy; endocrinology & metabolism |
| 7-9 | pharmacology & pharmacy; chemistry, medicinal; biotechnology & applied microbiology; cardiac & cardiovascular systems; health policy & services |
| 8-1 | business, finance; economics; management; business; operations research & management science |
| 8-2 | economics; management; business; operations research & management science; social sciences, mathematical methods |
| 8-3 | business, finance; economics; management; business; operations research & management science |
| 8-4 | economics; management; business; computer science, interdisciplinary applications; environmental studies |
| 8-5 | management; economics; business; business, finance; environmental sciences |
| 8-6 | business, finance; economics; management; business; computer science, information systems |
| 8-7 | economics; management; business; environmental studies; business, finance |
| 8-8 | business, finance; economics; management; business; operations research & management science |
| 8-9 | economics; business, finance; business; management; environmental sciences |
| 8-10 | economics; business, finance; management; business; environmental sciences |
| 9-1 | economics; environmental studies; energy & fuels; environmental sciences; green & sustainable science & technology |
| 9-2 | environmental sciences; economics; environmental studies; green & sustainable science & technology; business |
| 9-3 | environmental sciences; green & sustainable science & technology; economics; environmental studies; energy & fuels |
| 9-4 | environmental sciences; business; management; environmental studies; green & sustainable science & technology |
| 9-5 | environmental sciences; green & sustainable science & technology; environmental studies; engineering, environmental; economics |
| 9-6 | economics; environmental studies; management; environmental sciences; business |
| 9-7 | economics; business; environmental studies; management; environmental sciences |
| 10-1 | chemistry, multidisciplinary; pharmacology & pharmacy; crystallography; materials science, multidisciplinary; chemistry, medicinal |
| 10-2 | pharmacology & pharmacy; nanoscience & nanotechnology; materials science, multidisciplinary; physics, applied; polymer science |
| 10-3 | pharmacology & pharmacy; polymer science; medicine, research & experimental; biochemistry & molecular biology; chemistry, medicinal |
| 10-4 | pharmacology & pharmacy; nanoscience & nanotechnology; materials science, multidisciplinary; physics, applied; chemistry, medicinal |
| 10-5 | pharmacology & pharmacy; nanoscience & nanotechnology; materials science, multidisciplinary; physics, applied; materials science, biomaterials |
| 11-1 | computer science, interdisciplinary applications; computer science, information systems; chemistry, multidisciplinary; chemistry, medicinal; mathematical & computational biology |
| 11-2 | chemistry, multidisciplinary; computer science, information systems; computer science, interdisciplinary applications; information science & library science; chemistry, medicinal |
| 11-3 | computer science, information systems; computer science, interdisciplinary applications; chemistry, multidisciplinary; information science & library science; communication |
| 11-4 | chemistry, medicinal; chemistry, multidisciplinary; pharmacology & pharmacy; computer science, information systems; computer science, interdisciplinary applications |
| 12-1 | pharmacology & pharmacy; integrative & complementary medicine; chemistry, medicinal; medicine, general & internal; plant sciences |
| 13-0 | pharmacology & pharmacy; chemistry, medicinal; biochemistry & molecular biology; oncology; chemistry, organic |
| 14-1 | law; economics; telecommunications; engineering, electrical & electronic; information science & library science |
| 14-2 | management; business; economics; telecommunications; communication |
| 14-3 | economics; management; business; environmental studies; information science & library science |
| 15-1 | chemistry, medicinal; chemistry, organic; pharmacology & pharmacy; biochemistry & molecular biology; chemistry, multidisciplinary |
| 15-2 | chemistry, medicinal; biochemistry & molecular biology; pharmacology & pharmacy; chemistry, organic; chemistry, multidisciplinary |
